# Genetic determinants of the phenotype in a Swedish cohort of patients with hypertrophic cardiomyopathy

**DOI:** 10.1038/s41598-025-27238-9

**Published:** 2025-11-10

**Authors:** Kissopoulou Antheia, Fernlund Eva, Karlsson Jan-Erik, Green Henrik, Ellegård Rada, Gunnarsson Cecilia

**Affiliations:** 1 Department of Internal Medicine, Department of Health, Medicine and Caring Sciences, County Council of Jönköping, Linköping University, Linköping, Sweden; 2 Department of Biomedical and Clinical Sciences, Division of Pediatrics, Linköping University, Crown Princess Victoria Children´S Hospital, Linköping University Hospital, Linköping, and Pediatric Clinic Vrinnevi Hospital, Norrköping, Sweden; 3 Department of Clinical Sciences Lund, Lund University, Skane University Hospital, Pediatric Heart Center, Lund, Sweden; 4 Division of Clinical Chemistry and Pharmacology, Department of Biomedical and Clinical Sciences, Linköping University, Linköping, Sweden; 5 Department of Forensic Genetics and Forensic Toxicology, National Board of Forensic Medicine, Linköping, Sweden; 6 Department of Clinical Genetics, and Department of Biomedical and Clinical Sciences, Linköping University, Linköping, Sweden; 7 Centre for Rare Diseases in South East Region of Sweden, Linköping University, Linköping, Sweden

**Keywords:** Clinical genetics, Genotype, Medical genetics, Sequencing, Cardiology, Cardiovascular biology

## Abstract

Hypertrophic cardiomyopathy (HCM), the most common inherited cardiomyopathy, is characterized by phenotypic and genetic heterogeneity. The present study describes the genotype data of a Swedish cohort of patients with HCM, the largest genetics study on Swedish HCM patients to date. The primary aims of this study were to unravel the main genetic findings and explore genotype–phenotype associations in this HCM cohort. Longitudinal data on 225 unrelated HCM index patients from the Southeast health care region in Sweden from 2010 until 2021 were assessed retrospectively. Patients were 46 ± 15.5 years-old, 67.6% males. In the cohort, 172/225 (76.4%) had genetic testing, of whom, 65/172 (38%) were considered genotype positive (G +) for a pathogenic/ likely pathogenic variant, mainly in the two most common sarcomeric genes: *MYBPC3* (57%) and *MYH7* (34%). In 43% (74/172) of patients, no reportable variants were detected, classified as genotype negative (G-). In the remaining 33 patients (19%), variants of uncertain significance (VUS) were identified; this group was not included in the comparative analyses. Genotype positive patients (G +) were characterized by younger age (p = 0.010), higher prevalence of family history of HCM (p < 0.001), greater maximum left ventricle wall thickness (p = 0.03) and an increased incidence of sudden cardiac death (SCD) (p = 0.045). At first clinical screening, HCM was diagnosed in 28/65(43%) in the G + families and in 2/74 (2.7%) G-families (p < 0.001). Genotype-positive HCM patients differ with respect to age at presentation, family history of the disease, morphology, incidence of SCD and presence of HCM in their family members at first clinical assessment from genotype-negative patients. Genotype negative status in this HCM cohort, though, did not confer immunity from adverse complications.

**Supplementary Information:**

The online version contains supplementary material available at 10.1038/s41598-025-27238-9.

## Introduction

Hypertrophic cardiomyopathy (HCM) is a heritable cardiovascular disorder with a diverse natural history, characterized by unexplained left ventricular hypertrophy (LVH), defined as a maximum left ventricular wall thickness (MLVWT) ≥ 15 mm in adult patients and ≥ 13 mm in family members^1,2^. In paediatric patients, HCM diagnosis requires an LV wall thickness greater than 2 standard deviations above the predicted mean for body size (z-score > 2)^2^. Many patients with HCM experience adverse outcomes, including heart failure (HF), atrial fibrillation (AF) with a risk of stroke, ventricular tachycardia (VT) and even sudden cardiac death (SCD). In 1990, sequence analysis of a candidate gene revealed a pathogenic missense variant in the beta-myosin heavy chain gene, *MYH7* to be responsible for HCM^3^. As of 2019, up to eleven causative genes with over 1500 variants in genes encoding the thick or thin myofilament proteins of the sarcomere have been reported^4^. The diagnostic yield of genetic screening for identifying pathogenic variants in patients with HCM varies across studies, ranging from 30 to 63% while in some populations with known founder variants, the yield of genetic testing can be as high as 72%^4^.

Gene-specific or variant-specific associations, although potentially more useful for decision-making, are more inconsistent and more difficult to replicate. This has limited the clinical use of genetics for prognostication in HCM. However, its role in diagnosis and family screening is well established and cascade screening can identify or exclude individuals at risk of developing the disease^2^. Genetic testing allows the identification of genotype-positive relatives without left ventricular hypertrophy (LVH) and the reassurance and discharge of genotype-negative relatives. Cardiac evaluation of at-risk relatives enables early diagnosis and identification of those patients at high risk for SCD, which can be the first manifestation of the disease.

Some genotype–phenotype correlations in HCM have been replicated in recent work on large populations^5–7^, mostly focusing on a comparison between individuals with and without pathogenic variants in one of the main sarcomere protein causal genes. Previous investigators reported a higher risk for adverse HCM‐related outcome in patients with pathogenic sarcomere variants^6,8–10^. A recent systematic review and meta-analysis^11^ has highlighted that sarcomere variants are more frequent in women, and are associated with worse clinical characteristics and poor outcomes.

Only a few studies have systematically analyzed the genetic and phenotypic aspects of HCM in Swedish population^12,13^. Mörner et al.^13^ described the genetic landscape and phenotype of HCM in the northern Swedish population. In view of this background, the present study aimed at gaining further knowledge about hypertrophic cardiomyopathy in the southeast region in Sweden. In this work, the main aim was to characterize the status of the clinical use of genetic testing in HCM patients and explore genotype–phenotype associations in this Swedish cohort. Furthermore, this study aimed to evaluate whether first-degree relatives had clinical screening for HCM and/or genetic testing for a familial pathogenic variant, and the percentage of the screened first-degree relatives that were given a diagnosis of HCM based on first evaluation (after diagnosis in the proband).

## Materials and methods

Longitudinal data from 225 unrelated index patients with an established diagnosis of hypertrophic cardiomyopathy were retrospectively evaluated at the outpatient cardiogenetic clinics of County Hospital Ryhov in Jönköping and the tertiary center at University Hospital in Linköping, Sweden, between January 2010 and December 2021.Patients were identified through the electronic medical records system using the International Classification of Diseases, Tenth Revision (ICD-10) codes I42.1 (obstructive HCM) and I42.2 (other HCM)^14^. An initial search yielded 454 patients with these HCM-related ICD-10 codes. After thorough review of the medical records, 287 patients (63.3%) were confirmed to have primary HCM, while 167 patients (36.7%) were excluded due to alternative causes of left ventricular hypertrophy (LVH), most commonly hypertrophy secondary to hypertension (63.5%), as well as other cardiomyopathies (11.4%) and amyloidosis (9.6%).

**Fig. 1:**
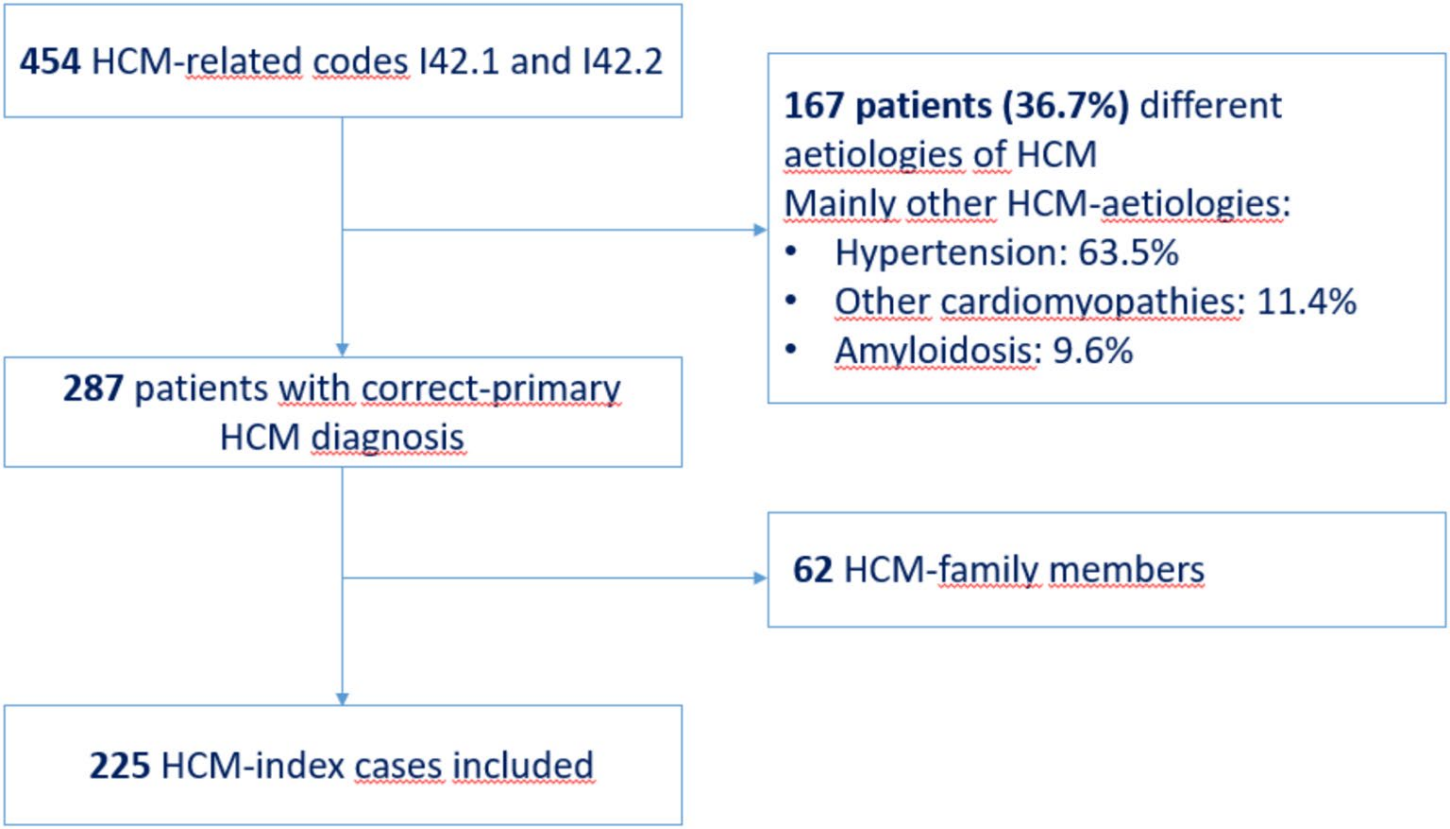
Flowchart of the study-population: Hypertrophic Cardiomyopathy (HCM) was identified by ICD-10 codes: I42.1 (obstructive HCM) and I42.2 (other HCM) codes from 2010 to 2021. From the 454 patients with these HCM-related codes I42.1 and I42.2, 287 (63.2%) patients were correctly classified as primary HCM and 167 patients (36.7%) had other aetiologies of HCM. Among the 287 HCM patients, 62 were family members of the index patient. Thus, the remaining 225 HCM-index patients were included in this study.

In accordance with the American College of Cardiology/American Heart Association (ACC/AHA) guidelines^15^, patients with left ventricular hypertrophy due to systemic or infiltrative disorders—such as amyloidosis, Fabry disease, sarcoidosis, RASopathies, and other phenocopies—were excluded from the study cohort. Although these conditions can present with a similar degree or pattern of hypertrophy, they differ from primary HCM in underlying pathophysiology, prognosis, and treatment. While the European Society of Cardiology (ESC) guidelines^2,16^ include such conditions within a broader classification of HCM-related disorders, the current analysis is based on the ACC/AHA definition^15^, which focuses specifically on primary sarcomeric or idiopathic forms of HCM. The complete list of categories of different aetiologies of HCM is presented in Supplementary Table 1. Among the 287 confirmed cases, 62 were family members of index patients, resulting in a final cohort comprising 225 unrelated index patients (Fig. 1). Although patients were identified based on HCM-related ICD-10 codes recorded between January 2010 and December 2021, many had received a diagnosis of HCM prior to this time period and continued follow-up at our cardiogenetic clinics. The date of first diagnosis, therefore, may precede the inclusion window, and follow-up duration reflects the time from the earliest documented HCM diagnosis to the most recent follow-up available in the medical records.

HCM was diagnosed morphologically using echocardiography in accordance with the European Society of Cardiology (ESC) guidelines^1,2,16^, defined by a non-dilated, hypertrophied left ventricle (LV) wall thickness of ≥ 15 mm, in the absence of other cardiac or systemic conditions that could explain the hypertrophy. Relatives to the HCM index patients older than age 18 years received a diagnosis of HCM in the presence of left ventricular hypertrophy ≥ 13 mm^2^.

Information obtained from the medical records included demographics, clinical parameters, family history for HCM or SCD, genetic testing, and pharmacological and non-pharmacological treatments. Regarding the echocardiographic measurements, the extent of left ventricular hypertrophy was measured from the 2D parasternal axis view at the basal, mid-LV and apical plane. Left ventricular ejection fraction, left atrial size and mitral regurgitation were measured as previously described. Maximal wall thickness was defined as the greatest measured at any left ventricular segment. The obstructive phenotype due to the systolic anterior motion (SAM) of the mitral leaflets was defined as left ventricular outflow peak gradient more than 30 mmHg at rest or provoked peak gradient more than 30 mmHg (Valsalva maneuver or standing) using continuous wave Doppler. Most of the cardiac magnetic resonance CMRs were performed using 3.0 Tesla CMR scanner. LV measures of geometry and function were analyzed using standardized protocols. Myocardial T1 mapping was used to assess for diffuse myocardial fibrosis, and late gadolinium enhancement (LGE) to assess myocardial fibrosis content.

The study protocol was approved by the Regional Ethics Committee in Linköping (Dnr 2016/389‐31), and all participants provided informed consent prior to enrollment. The study adheres to the ethical principles of the 1975 Declaration of Helsinki. Two patients presented with sudden cardiac death as their first symptom; in these cases, diagnosis was confirmed post-mortem through autopsy measurements. Genetic testing was also performed post-mortem, and consent for participation and genetic analysis was obtained from first-degree relatives.

Clinical, genetic, and outcomes data on 225 patients with HCM are presented as frequency (and percentage) for non-continuous variables and mean ± SD for continuous variables where appropriate. Student’s t test was used for the comparison of continuous variables. A P-value < 0.05 was considered statistically significant. Categorical variables were expressed as frequencies and percentages and were compared using Chi-square test or Fisher’s exact test for small samples relatively. Data analysis was performed using SPSS version 27.

### Genetic testing and variant classification

Genetic testing was performed on the index case using the clinically approved cardiomyopathy gene panel available at the time of referral. These panels varied over time and included both sarcomeric and non-sarcomeric genes associated with cardiomyopathy. All patients provided informed consent for genetic testing and analysis.

Prior to April 2018, 85 patients were tested using a targeted cardiomyopathy gene panel covering a defined set of genes, targeted sequencing was used, covering the following genes:

*ABCC9, ACTC1, ACTN2, ANKRD1, BAG3, CAV3, CSRP3, DES, DMD, DSC2, DSG2, DSP, EMD, FHL1, FHL2, GLA, JUP, LAMP2, LDB3, MYBPC3, MYH6, MYH7, MYL2, MYL3, MYLK2, MYOZ2, NEBL, NEXN, PKP2, PLN, PRKAG2, RBM20, SCN5A, TAZ, TCAP, TGFB3, TMEM43, TMPO, TNNC1, TNNI3, TNNT2, TTR, VCL.*

From April 2018 onwards, 87 patients underwent exome sequencing with analysis using a virtual panel based on the Genomics England PanelApp for cardiomyopathy, which expanded the number of genes tested^17^. Initial variant interpretation was performed by experienced clinical staff at the time of patient referral. As new evidence and guidelines that may affect variant interpretation emerge over time, the variants were re-evaluated during preparation of this manuscript. Variant interpretation was performed using QCI (Qiagen). Variants were assessed according to American College of Medical Genetics and Genomics (ACMG) guidelines^18^. For genes with curated criteria specifications by ClinGen Variant Curation Expert Panels listed in the ClinGen Criteria Specification Registry (https://clinicalgenome.org/) (*ACTC1, MYL2, MYL3, MYH7, MYBPC3, TNNI3, TNNT2*), application of ACMG criteria was performed according to these guidelines.

Genetic variants classified as pathogenic or likely pathogenic (P/LP), based on ACMG guidelines, were included in the analysis and reported (as detailed in Supplementary Table 2). Regarding variants of uncertain significance (VUS), reporting practices have evolved over time, generally becoming more restrictive^19^. Throughout the study period, inconsistencies in VUS reporting, combined with the fact that VUS do not currently guide clinical management^16,19^, led to the decision to exclude VUS variants from the genetic analysis and from genotype-positive classification. VUS variants in sarcomeric genes were not considered pathogenic or likely pathogenic unless supported by additional strong evidence. VUS variants in genes not clearly associated with hypertrophic cardiomyopathy (e.g., *DSP, PKP2, COL6A1, SGCD, RYR2, MYPN*) were identified and reported in the Supplementary material but were not included in the gene-negative group or primary analyses. Although VUS variants in these non-HCM-related genes could be considered part of the genotype-negative group, they were reported separately to avoid potential misclassification and maintain clear group definitions. This approach was chosen due to the uncertain clinical relevance of these variants to HCM, avoiding misclassification and potential bias.

Patients were categorized into three groups based on genetic results:

(1) Genotype-positive – carriers of P/LP variants in any cardiomyopathy-associated gene;

(2) VUS-only – carriers of only variants of uncertain significance;

(3) Genotype-negative – no reportable variants.

Only genotype-positive and genotype-negative groups were included in comparative analyses. Patients who carried only VUS variants were not included in either group and were therefore excluded from all comparative analyses. However, the identified VUS variants are listed in Supplementary Table 3.

While many variants were identified in well-established sarcomeric genes, broader cardiomyopathy panels —particularly after 2018— were utilized to account for phenotypic overlap and genetic heterogeneity. All pathogenic or likely pathogenic (P/LP) variants associated with HCM were included in the analysis, regardless of the strength of gene-disease association. Eight sarcomeric genes *(MYH7, MYBPC3, TNNT2, TNNI3, ACTC1, TPM1, MYL2, MYL3)* are currently considered to have definitive evidence for HCM^20^. However, genes such as *ALPK3* and *FLNC*, which are widely used nowadays in clinical panels and supported by strong or emerging evidence, were also included in the main analysis when relevant pathogenic variants were identified.

Statistical analyses compared genotype-positive and genotype-negative patients. Kaplan–Meier survival analyses were conducted within the genotyped hypertrophic cardiomyopathy subgroup, assessing overall mortality, cardiovascular mortality, SCD, and heart failure progression between the two groups. Cardiovascular mortality included deaths directly related to the primary disease, such as SCD, stroke-related, and heart failure-related deaths. Complications and adverse events included heart failure, heart transplantation, stroke, appropriate ICD shocks, myocardial infarction, embolism, death, SCD, sustained ventricular tachycardia (VT), and resuscitated cardiac arrest.

## Results

### Baseline demographic, clinical and imaging characteristics of hypertrophic cardiomyopathy patients at index evaluation

In all, 225 unrelated and consecutive patients with HCM were studied. Mean follow-up time was 16.4 ± 10.5 years (0–54 years). Mean age at diagnosis was 46 ± 15.5 years. Male patients (67.6%) were diagnosed with HCM at a younger age than women (mean age of men: 43.8 ± 14.5 whereas female: 51.4 ± 16.37, p < 0.001). Baseline characteristics and descriptive information on outcomes are summarized in Table 1, 2 and 3.

**Table 1:** Baseline demographic and clinical characteristics of hypertrophic cardiomyopathy patients at index evaluation.

**Baseline characteristics**	
Swedish ethnicity	192/225 (85.3%)
Age at diagnosis, mean (SD, min max)	46 ± 15.5 (6—77)
Sex, male	152/225 (67.6%)
Hypertension	102/225 (45.3%)
Angina*	33/225 (14.7%)
Diabetes	26/225 (11.6%)
Smoking	80/225 (35.6%)
**Reason for diagnosis**	
Symptoms	173/225 (76.9%)
Check-up/Incidental	52/225 (23.1%)
History of pericarditis/myocarditis	8/225 (3.6%)
**Presentation**	
Family history of HCM	55/225 (24.4%)
Family history of SCD	53/225 (23.6%)
Initial ECG (LVH criteria**)	210/225 (93.3%)
Predominant Symptom at Initial Evaluation*	
Dyspnea	78 (34.7%)
Chest pain	48 (21.3%)
Palpitations	33 (14.7%)
NYHA class III/IV	6 (2.7%)
Syncope	10 (4.4%)
Resuscitated cardiac arrest	4 (1.8%)
SCD	2 (0.9%)
Tiredness	11 (4.9%)
No symptoms at all	33 (14.7%)
**Clinical Signs**	
Abnormal SBP response to exercise	42 (18.7%)
Murmur	148/225 (65.8%)

Footnotes:

* Angina refers to patients with documented ischemic heart disease; chest pain refers to non-anginal discomfort reported at presentation.

** ECG findings consistent with LVH include increased QRS voltage, repolarization abnormalities (ST-T changes), and pathological Q waves.

*Only the most predominant symptom per patient was recorded.

HCM: hypertrophic cardiomyopathy, SCD: sudden cardiac death, ECG: electrocardiogram, SD: standard deviation, NYHA class: New York Heart Association functional class, SBP: Systolic blood pressure , LVH: left ventricular hypertrophy.

**Table 2:** Baseline imaging characteristics of hypertrophic cardiomyopathy patients at index evaluation.

**Initial echocardiography**	
Max wall thickness mm, mean (SD, min max)	20.7 ± 4.4 (13—46)
Apical hypertrophy	50/225 (22.2%)
EF (%)	60.8 ± 8.5 (20—81)
Obstructive type	108/225 (48%)
Peak LVOTgradient (mmHg), mean (SD, min max)	55 ± 60.7 (4—345)
LVOT gradient > 30 mmHg	105/225 (46.6%)
LA, size (mm), mean (SD, min max)	46 ± 5.8 (30—62)
Apical aneurysm	12/225 (5.3%)
**CMR**	
CMR performed	100/225 (44.4%)
Late gadolinium enhancement	82/100 (82%)

SD: standard deviation, EF: Ejection fraction, LVOT: Left ventricular outflow tract, LA: left atrium, CMR: Cardiovascular magnetic resonance imaging.

**Table 3:** Follow-up data, events, interventions, complications and mortality of the Hypertrophic cardiomyopathy (HCM) cohort.

**Follow-up**	
Atrial fibrillation	87/225 (38.7%)
NSVT	72/225 (32%)
Syncope	43/225 (19.1%)
**Interventions**	
Myectomy	8/225 (3.6%)
Alcohol septal ablation	37/225 (16.4%)
Pacemaker	32/225 (14.2%)
ICD	60/225 (26.7%)
Heart transplantation	4/225 (1.8%)
**Complications**	85/225 (37.8%)
Stroke	22/225 (9.8%)
Myocardial infarction	19/225 (8.4%)
Heart Failure	32/225 (14.2%)
End-stage Heart Failure	6/32 (18.75%)
Arterial Embolism	2/225 (0.9%)
Appropriate ICD discharges	6/60 (10%)
Resuscitated cardiac arrest	8/225 (3.6%)
**Mortality**	
Overall mortality	30/225 (13.3%)
Cardiovascular mortality	17/225 (7.6%)
SCD	10/225 (4.4%)
Death from heart failure	5/225 (2.2%)
Death from stroke	2/225 (0.9%)
**Medications**	
None	13 (5.8%)
Betablockers	162 (72%)
Verapamil	7 (3.1%)
Diltiazem	4 (1.8%)
Disopyramide	4 (1.8%)
Betablockers + Amiodarone	7 (3.1%)

NSVT: Non-sustained ventricular tachycardia, SCD: Sudden cardiac death, ICD: Implantable cardioverter-defibrillator.

Asymmetrical septal hypertrophy was the most common phenotype in this cohort, followed by apical hypertrophy pattern (22.2%) as identified by echocardiography and confirmed by CMR when available. Apical hypertrophic cardiomyopathy (ApHCM) is a relatively rare morphological variant of HCM, defined by unexplained left ventricular hypertrophy predominantly localized to the apex of the left ventricle. On imaging, ApHCM may present with a characteristic “spade-like” configuration of the left ventricular cavity. An apical aneurysm was detected in 5.3% of patients within the apical phenotype group. The apical group included both “pure” ApHCM, characterized by isolated asymmetric hypertrophy confined to the apex, and “mixed” ApHCM (distal-dominant form), in which apical hypertrophy coexisted with interventricular septal thickening.

Mean maximal wall thickness in the HCM cohort was 20.7 ± 4.4 mm with high‐risk massive hypertrophy (≥ 30 mm) in eleven patients (5%). Resting or provoked left ventricular outflow obstruction was present in 108 (48%) patients. Cardiovascular magnetic resonance imaging (CMR) was performed in 100 patients (44.4%) in the cohort. Interestingly, 82/100 of them had late gadolinium enhancement (LGE) uptake.

Positive family history of the disease was present in 24.4% of patients indicating a familial inheritance, whereas positive family history for SCD was seen in 23.6%. Most of the patients (76.9%) were diagnosed due to symptoms of the disease and 23.1% were diagnosed incidentally. At the initial evaluation 93.3% (n = 210) had pathological ECG consistent with left ventricular hypertrophy, including increased QRS voltage and/or repolarization abnormalities. The patients with normal ECGs (13/225) at the initial evaluation were older (58 ± 11 vs 45 ± 16, p = 0.001), had less maximal wall hypertrophy (18.7 ± 2.5 vs 21 ± 4, p = 0.014) and experienced less adverse events; neither of them presented with SCD nor developed heart failure. In the two patients, who initially presented with SCD, ECGs were missing.

### Events, complications and interventions of the HCM cohort

Atrial fibrillation (AF) was present in 38.7%, whereas non-sustained ventricular tachycardia occurred in 32% of the patients. There was no significant difference between AF and sex (p = 0.66) neither for malignant arrhythmias and sex (p = 0.2). A total of 43 (19.1%) patients reported a history of syncopal episodes and eight had a resuscitated cardiac arrest.

ICDs were implanted in 26.7% of the patients and 10% of those had appropriate ICD discharges. Beta-blockade therapy as monotherapy was being administrated in 72% patients.

During follow-up, 85 (37.8%) patients, experienced at least one HCM-related complication; summarized in Table 3. Heart failure was reported in 32 (14.2%) HCM patients and six of them even developed end-stage whereas four had undergone heart transplantation. During surveillance, a total of 13.3% (30/225) of patients died. Specifically, 7.6% of the HCM patients died from cardiovascular causes, including 2.2% from heart failure and 4.4% patients (10/225) from sudden death. 3.6% had undergone myectomy whereas alcohol septal ablation was performed in 16.4%. All the events and the complications of the disease in the population are displayed in Table 3.

### Genetic testing and results of the HCM cohort

Among the 225 hypertrophic cardiomyopathy (HCM) index patients recruited, 172 (76.4%) underwent genetic testing, while 53 did not. There was not a significant difference between the frequency of tested males (115 in 152, 75.6%) compared to females (57 in 73, 78%). The main reasons for not performing genetic testing included the absence of a test request by the referring cardiologist (~ 17.3%) and patient reluctance or refusal (14 of 174 patients invited).

To address potential selection bias, clinical characteristics were compared between the tested and non-tested groups, revealing no significant differences in age, sex, maximum left ventricular wall thickness, left atrial diameter, or presence of hypertension. However, there was a trend toward higher left ventricular outflow tract (LVOT) gradients in the untested group (p = 0.068). Significant differences were observed in some clinical features:

- Patients who did not undergo genetic testing had a higher prevalence of atrial fibrillation (p = 0.002) and complications related to HCM (p = 0.004).
- Death and heart failure were significantly more frequent in patients who did not undergo genetic testing (p < 0.001 for both).
- Patients with a history of sudden cardiac death (SCD) and those with a family history of HCM were more likely to have undergone genetic testing (p = 0.015 and p = 0.007, respectively).
- Fibrosis on cardiac magnetic resonance imaging (CMR) was more common in the genetically tested group (p = 0.024).

These findings suggest that patients who did not undergo genetic testing might have experienced more severe or acute clinical events, possibly limiting access to genetic testing, or that genetic testing was performed earlier in more stable patients.

Of the 172 patients, 65 (38%) were considered genotype positive (G +) with a pathogenic/P or likely pathogenic/LP variant, most common in two sarcomeric genes: *MYBPC3* (57%) and *MYH7* (34%) and less frequently: *ACTC1* (1.5%), *ALPK3* (1.5%), *FLNC* (3%), and *MYL2* (3%) genes. In approximately 6% of patients there was more than one pathogenic variant detected and 33 patients (19%) had variants of unknown significance (VUS) (Supplementary Table3).

A total of 37 probands were found to carry pathogenic or likely pathogenic variants in the *MYBPC3* gene, involving 17 different unique variants. Among these, the c.2490dup variant (also described as NM_000256.3:c.2490dup, which leads to a frameshift and predicted premature truncation of the protein, p.(His831fs)) was the most frequently identified variant, present in 10 probands. This duplication variant is known to cause a frameshift resulting in a truncated protein product likely leading to loss of normal function of *MYBPC3*, which is a well-established mechanism in HCM. The high frequency of this particular variant in the cohort suggests it may be a common founder variant or a hotspot for genetic changes in this population. Other frequent *MYBPC3* variants included c.710A > C in 6 probands and c.2373dup in 5 probands, while the remaining 14 variants were each found in one or two probands. For the *MYH7* gene, 22 probands carried pathogenic or likely pathogenic variants distributed across 13 unique variants, with the most common being c.1988G > A, identified in 4 probands. Variants in other genes such as *ACTC1, MYL2, ALPK3*, and *FLNC* were infrequent and typically unique to single probands (Supplementary Table 2.).

In 43% (74/172) of patients no reportable variants were detected, (genotype negative, G-). In eleven of the 74 (15%) HCM patients without any identified genetic variant there was a family history of HCM and SCD.

According to current guideline recommendations^2,15,16^, all index patients with HCM should be offered family screening irrespective of the results of genetic testing. In this cohort, 95.6% of the HCM probands (215/225) had their family members under clinical cascade screening, regularly followed-up with ECG, Echocardiogram, Holter, and exercise test according to guidelines. Family members with a positive genotype detected in the proband, were offered genetic testing and if the familial pathogenic genetic variant was not detected, they were discharged from any clinical surveillance. At the initial clinical evaluation, the relatives who fulfilled HCM diagnostic criteria appeared in 17.2% (37/215) of the families investigated. From these 37 families, 34 had their index-proband genetically tested, where 28/34 (82.3%) had a P/LP variant, and 4 (11%) VUS, only in two of them there were no genetic variants detected.

Taking into consideration the current guidelines^2,15,16^, the first degree relatives of all the probands with a P/LP variant were offered genetic counselling and genetic testing, thus in all of the 65 genotype positive families, at least one relative underwent genetic investigation. In 28 of the 65 (43%) genotype positive families, at least one family member depicted signs of HCM at initial clinical evaluation whereas only in 2 /74 (2.7%) genotype negative families. The results of the genetic testing of this HCM cohort are presented in Table 4 and the complete list of all P/LP variants is presented in Supplementary Table 2.

**Table 4:** Genetic Testing and Results of the Hypertrophic cardiomyopathy (HCM) cohort.

Genetic Testing	172/225 (76.4%)
Reluctant to testing	14/225(6.2%)
**Result of Genetic test**	
Pathogenic/Likely pathogenic	65/172 (37.8%)
No mutation found	74/172 (43%)
Variants of unknown significance (VUS)	33/172 (19%)
**Genetic Variants (Pathogenic/Likely Pathogenic)**	
*MYBPC3*	37/65 (57%)
*MYH7*	22/65 (34%)
Others (*ACTC1, MYL2, ALPK3, FLNC)*	6/65 (9%)
Double mutations	4/65 (6%)
**Family screening**	
Clinical Screening	215/225 (95.6%)
HCM phenotype in families at initial clinical screening	37/215 (17.2%)
HCM phenotype in G + families at initial clinical screening	28/65 (43%)
HCM phenotype in G- families at initial clinical screening	2/74 (2.7%)

G + : genotype positive, G-: genotype negative.

### Genotype associations with clinical parameters and events

The average age for onset of HCM for the genotype-positive individuals was 41 ± 16.3 years, which was significantly younger than the onset age (48 ± 14.8 years, p = 0.01) for genotype-negative individuals. This study illustrated a higher proportion of individuals with a family history of HCM among the genotype-positive patients (52.3% vs. 14.8%; p < 0.001). In addition, the proportion of patients with a family history of SCD was significantly higher in the genotype-positive group than in the genotype-negative group (40% vs 16.2%; p = 0.001).

There were significantly fewer patients with hypertension in the genotype-positive group (24.6% vs 60.8%; p < 0.001) in the genotype-negative group.

In terms of the echocardiographic parameters, the mean maximal wall thickness was higher in genotype-positive patients (22 ± 5 vs 20 ± 3.5 mm, p = 0.03) whereas the mean left atrial diameter was higher in genotype-negative patients (47 ± 5.7 vs 44 ± 5.6 mm, p = 0.012). A lower prevalence of an apical pattern of hypertrophy (6.1% in vs 35.1%, p < 0.001) was observed in genotype-positive patients. No other echocardiographic parameters were significantly associated with genotype-status. There was no significant difference in obstruction profile, neither any significant associations with cardiac magnetic resonance imaging (CMR) parameters, fibrosis and genotype were observed as shown in Table 5.

**Table 5:** Genotype associations with baseline demographic and clinical parameters.

	**Genotype G- (n = 74)**	**Genotype G + (n = 65)**	**P-values** **G + vs G-**
Female, n (%)	25 (34)	21 (32.3)	0.85
Age at diagnosis, Mean ± SD	48 ± 14.8	41 ± 16.3	**0.01**
Hypertension	45 (60.8)	16 (24.6)	** < 0.001**
Diabetes	13 (17.5)	1 (1.5)	**0.001**
Family history of HCM	11 (14.8)	34 (52.3)	** < 0.001**
History of SCD	12 (16.2)	26 (40.0)	**0.001**
Phenotype in the family	2 (2.7)	28 (43)	** < 0.001**
Maximal LVWT mm, Mean ± SD	20 ± 3.5	22 ± 5	**0.03**
LVOT max mmHg, Mean ± SD	53 ± 64	39 ± 43	0.13
Obstructive type	35 (47.2)	26 (40.0)	0.39
Apical type	26 (35.1)	4 (6.15)	** < 0.001**
Left atrium mm, Mean ± SD	47 ± 5.7	44 ± 5.5	**0.012**
LVEF %, Mean	60.5 ± 7.7	62 ± 6.9	0.25
Fibrosis on CMR	33 (84.6)	21 (84)	0.95
Pacemaker	9 (12,1)	8 (12.3)	1
ICD	20 (27)	21 (32.3)	0.39
Myectomy	2 (2.7)	3 (4.9)	0.66
Alcohol septal ablation	15 (20.2)	9 (13.8)	0.32

P-value between Genotype negative G- vs Genotype positive G + HCM patients, variants of unknown significance (VUS) excluded.

SD: standard deviation, SCD: sudden cardiac death, LVWT: Left ventricular wall thickness, LVOT: Left ventricular outflow tract, LVEF: Left ventricular ejection fraction, CMR: Cardiovascular magnetic resonance imaging, HCM: Hypertrophic cardiomyopathy.

**Fig. 2:**
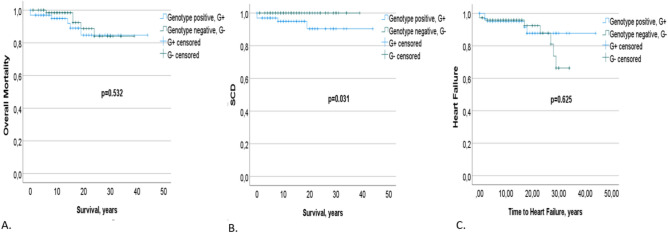
Kaplan–Meier survival analyses. Kaplan–Meier survival analyses were performed in the genotyped hypertrophic cardiomyopathy (HCM) subset = 172 patients. A. There was no significant difference in overall mortality between genotype positive G + vs genotype negative G-, p = 0.532; from the 30 patients who died: 6 had a pathogenic or LP variant, 3 had a variant of unknown sigificance (VUS), 5 didn’t have any genetic variant and in the rest 16 the genotype status was unknown. B. Genotype positive, G + patients had a higher incidence of SCD, p = 0.031; Totally 10 HCM patients had SCD, 4 of them had a pathogenic or LP variant and one had (VUS). In the rest 5 patients genotype status was unknown. C. There was no significant difference regarding the risk of developing heart failure (HF) between G + vs G-, p = 0.625, 17 of the 32 patients that developed heart failure were genotyped; 5 had a pathogenic/LP variant, 4 a VUS and 8 didn’t have any detected variant. A to C: blue: genotype-positive G + ; green: genotype-negative G- individuals; Survival time and time to Heart Failure: years.

Genotype-positive status was associated with SCD (p = 0.045) shown in Table 6. In survival analysis, a genotype-positive status was associated with SCD (log-rank p = 0.031). Genotype (G + or G −) though, was not associated with all‐cause and HCM‐related mortality (Fig. 2). There were no significant differences in HCM-related complications between G + and G − ; more specifically, neither group had a more increased risk of developing heart failure (HF), atrial fibrillation, embolic stroke, myocardial infarction, appropriate ICD shocks, embolic complications or ventricular arrhythmias (Table 6).

**Table 6:** Genotype associations with outcomes and events.

	**Genotype G- (n = 74)**	**Genotype G + (n = 65)**	**P-values** **G + vs G-**
All-cause death N (%)	5 (6.7)	6 (9)	0.76
Cardiovascular death, N (%)	3 (4)	4 (6)	0.70
Sudden cardiac death, SCD, N (%)	0	4 (6)	**0.045**
Atrial Fibrillation	29 (39)	18 (27)	0.15
VT	23 (30)	21 (34)	0.62
ICD	20 (27)	21 (32)	0.39
Overall Composite/Complications	23 (31)	22 (34)	0.73
Stroke	7 (9.5)	4 (6)	0.54
Myocardial Infarction	8 (11)	4 (6)	0.38
Heart Failure	8 (11)	5 (7.7)	0.57
Appropriate ICD shock	0	2 (9.5)	0.21
Resuscitated cardiac arrest	1 (1.4)	3 (4.6)	0.34
Emboli	0	1 (1.5)	0.47
Transplantation	0	1 (1.5)	0.47

P-value between Genotype negative G- vs Genotype positive G + HCM patients.

SCD: sudden cardiac death, VT: ventricular tachycardia, ICD: Implantable cardioverter-defibrillator.

There were no significant differences in HCM patients with pathogenic variants in *MYBPC3* vs *MYH7*, regarding age of onset (*MYBPC3*: 42.86 ± 17.1, *MYH7*:38.41 ± 15.3, p = 0.3), maximal wall thickness (*MYBPC3*: 22.73 ± 4.9 vs *MYH7*: 20.82 ± 4.9, p = 0.15), obstructive profile (*MYBPC3*: 18/37, 48%, *MYH7*: 7/22, 32%, p = 0.2), adverse events (*MYBPC3*: 13/37, 35%, *MYH7*: 8/22 ,36% p = 0.9); heart failure (*MYBPC3*: 4/37, 10%, *MYH7*:1/22, 4.5%, P = 0.4), stroke ( *MYBPC3*: 2/37, 5%, *MYH7*: 1/22, 4.5%, p = 0.9) and SCD ( *MYBPC3*: 2/37, 5% vs *MYH7*: 2/22, 9%, p = 0.6).

## Discussion

This present study of a Swedish cohort of patients with Hypertrophic Cardiomyopathy, is the largest HCM genetic study in Sweden. The study provides a detailed contemporary assessment of the clinical profile, genetic background, management strategies and outcomes of HCM. Approximately more than one-third of the sample had a miscoded HCM diagnosis from a cardiology/internal medicine facility, which underlines the diagnostic difficulties in a real-world setting. 36.7% of patients diagnosed as HCM in our cohort had different aetiologies of HCM and were excluded. Basic et al.^21^ highlighted in their study that, in hospital records from > 600 patients in western Sweden during a period of 20 years, the overall accuracy of cardiomyopathy diagnoses was high. The validity of DCM and HCM in three hospitals in western Sweden has been previously reported to be 85.5% and 87.5%, respectively. The large number of medical records scrutinized as well as the studied patients covering a large part of western Sweden, including the university tertiary referral hospital center, could have contributed to this high validity in diagnoses. However, a study by Magnusson et al.^22^ found a validity of only 68.2% for HCM patients , where only HCM patients from a small regional hospital in north Sweden were included. The most common reason for miscoded HCM in this cohort was hypertension. Nonetheless, concomitant hypertension was observed in 102 of 225 HCM patients (45.3%) (Table 1).

Hypertension was used as an exclusion criterion specifically to rule out cases in which left ventricular hypertrophy was primarily attributable to hypertensive heart disease rather than HCM. However, the presence of hypertension in a substantial proportion of confirmed HCM cases reflects the clinical reality that many individuals with true HCM also present with coexisting hypertension that does not fully account for the degree or pattern of hypertrophy. Previous studies^23^ have similarly reported a high prevalence of coexisting hypertension among HCM patients, typically ranging from 30 to 50%. Consequently, hypertension remains a common comorbidity across the age spectrum in HCM populations. The overlap between HCM and hypertensive cardiomyopathy presents diagnostic challenges, as both conditions can result in similar structural myocardial changes. However, hypertrophy in HCM tends to be more pronounced, asymmetric, and often associated with dynamic left ventricular outflow tract obstruction (LVOTO). Accurate differentiation requires a comprehensive assessment including clinical history, imaging features, and, where indicated, genetic testing^24^. In this study, among the 65 patients with pathogenic or likely pathogenic variants, only 16 (24.6%) had coexisting hypertension. Patients with both HCM and hypertension were significantly older at the time of diagnosis compared to those without hypertension (53 ± 11 vs. 39.9 ± 16 years, p < 0.001). Additionally, the prevalence of LVOTO was significantly higher in the hypertensive subgroup (58/102 vs. 50/123, p = 0.016), suggesting that coexisting hypertension may contribute to or exacerbate dynamic obstruction in a subset of HCM patients. These observations are in line with findings from previous studies^25^.

There are limited studies of Swedish HCM cohorts that have systematically included genetic background. Mörner et al.^13^ firstly described the genotypes and associated phenotypes related to HCM in northern Sweden. From the 46 individuals diagnosed with HCM, pathogenic genetic variants were found in 13 individuals. Genetic variants in the cardiac myosin-binding protein C gene (*MYBPC3*) were the most common ones found in northern Sweden, which are also found in this larger Swedish HCM study from the southeast region in Sweden. In approximately 6% of patients there was more than one pathogenic variant detected and these patients had a more severe clinical outcome supporting the literature data where it is reported that the number of genetic hits are associated with earlier and more severe disease expression^26^. One patient was homozygous of a *MYBPC3*-pathogenic variant and developed severe heart failure that resulted in heart-transplantation, as described earlier^27^. In this study, genetic testing was performed using a broad gene panel uniformly applied to all patients without subdivision into sarcomeric and non-sarcomeric gene categories. Therefore, comparative analyses based on these groupings were not conducted. Stratifying patients by sarcomeric versus non-sarcomeric variants could reveal additional clinical correlations and enhance genotype–phenotype understanding. Future studies designed with this approach may provide valuable insights into the heterogeneity of HCM. Genetic analysis confirmed that the majority of pathogenic variants were identified in well-established sarcomeric genes, predominantly *MYBPC3* and *MYH7*, consistent with the known genetic etiology of HCM. Additional sarcomeric gene variants were detected less frequently in *ACTC1* and *MYL2.* Notably, cytoskeletal gene variants such as those in *FLNC*, as well as sarcomere-related variants in *ALPK3,* were rare and found in only three patients within the cohort. These findings emphasize the predominance of sarcomeric variants in HCM while also highlighting the presence of less common variants in cytoskeletal and sarcomere-related genes that may contribute to disease heterogeneity.

This study has confirmed previously reported associations of genotype-positivity^5–7,28^ with a younger age at presentation, family history of HCM and SCD, absence of hypertension, increased prevalence of asymmetric compared to apical HCM and greater maximal wall thickness. A recent metanalysis^11^ revealed that sarcomeric genetic variants are more frequent in women, and are associated with worse clinical characteristics and poor outcomes. However, there were no significant differences in HCM patients with pathogenic variants in *MYBPC3* vs *MYH7*, regarding age of onset, imaging profile and adverse HCM events, the low number of events in each group limited the possibility of any multivariable analysis.

Similar to published studies^3,29^, there was not any pathogenic genetic variant detected in 43% of patients and surprisingly, in 15% of these HCM patients without any reportable genetic variant there was a family history of HCM and SCD. In the present study, whole exome sequencing was not routinely performed for genotype-negative cases, as targeted gene panels represented the standard clinical approach during the study period. Testing focused on well-established HCM-related genes, with expansion beyond these panels limited before 2018 due to the targeted sequencing design. Following the adoption of exome-based sequencing, all genes with established associations to cardiomyopathy were included. Expanding analysis to genes without established cardiomyopathy associations was beyond the study’s scope and not clinically informative, as such variants currently lack evidence to impact patient management^20^.

These genotype negative HCM patients were older at diagnosis, were more hypertensive and 31% of them experienced at least one HCM adverse events. There were three deaths related to HCM but not as SCD, one patient had a resuscitated cardiac arrest and eight patients developed heart failure (Table 6). Ventricular tachycardias were seen in 30% of them and atrial fibrillation in 39%. These data underscore an important principle in HCM in which genotype‐negative status does not necessarily convey a benign prognosis. Our data are consistent with the recently published study by Maron et al.^30^, where they highlight that genotype (G + or G −) was not a predictor of clinical course in HCM.

Atrial fibrillation is the most common arrhythmia associated with the disease with a reported prevalence of 38.7% among this HCM cohort. There was no significant difference between sex, neither between Genotype + and Genotype – HCM patients. However, the mean left atrial diameter (LA) was higher in genotype-negative patients (47 ± 5.7 vs 44 ± 5.5 mm, p = 0.012). This could be explained that genotype – patients were older at diagnosis and had several comorbidities such hypertension (G- vs G + p < 0.001, diabetes (G- vs G + , p = 0.0014) that could contribute to the atrial dilatation (Table 5)^31^. Both conditions are known contributors to diastolic dysfunction and increased left ventricular filling pressures, ultimately promoting LA dilatation. Chronic hypertension increases left ventricular afterload and impairs diastolic function, resulting in elevated filling pressures and subsequent LA dilation^32^. Obesity adds further hemodynamic and metabolic strain through mechanisms such as increased blood volume, systemic inflammation, and neurohormonal activation. Similarly, diabetes is associated with microvascular dysfunction, myocardial fibrosis, and diastolic impairment—all of which can promote progressive LA enlargement. These comorbidities likely play a synergistic role in modifying the HCM phenotype among genotype-negative patients, with LA enlargement representing a marker of both intrinsic cardiomyopathy and secondary remodeling due to systemic disease. This highlights the need for aggressive management of modifiable risk factors in this population to mitigate LA enlargement and its clinical consequences, including atrial fibrillation and thromboembolic risk.

Most patients in this cohort with HCM LVOT obstruction responded well to medical therapy, which likely reduced the need for invasive interventions. Among those who underwent invasive treatment, 3.6% had septal myectomy, while a higher proportion (16.4%) underwent alcohol septal ablation. Notably, patients treated with ablation were slightly older (48.7 ± 11.5 years) compared to those undergoing myectomy (43 ± 12 years). This difference may reflect patient selection and institutional preferences. Many centers may favor septal ablation due to its less invasive nature, broader accessibility, and lower immediate surgical risk, particularly in older patients or those with comorbidities. In contrast, septal myectomy requires specialized surgical expertise and facilities, which are often limited to select high-volume tertiary centers. This disproportion should be taken into account when interpreting treatment outcomes and may represent a selection bias within the study cohort. Nguyen et al.^33^ reported no significant differences in overall survival between patients undergoing myectomy or alcohol septal ablation; however, septal myectomy was associated with superior freedom from reintervention and more effective early and long-term reduction of the left ventricular outflow tract gradient.

Over follow‐up, 30 (13.3%) patients died, 17 from HCM‐related causes; 5 from heart failure, 2 from stroke and 10 from SCD. Although SCD is the most devastating event of the disease, it is relatively rare and affects mainly younger individuals. In our cohort 10 HCM patients had SCD (mean age 45.20 ± 13.4, and eight of them were male), two of them had SCD as initial presentation. Genetic testing was only performed in 5/10 and two had pathogenic variants in *MYBPC3* and two in *MYH7* and one had a VUS in *MYBPC3*. Post-mortem genetic testing is of paramount importance to detect the genetic aetiology of SCD and enable cascade screening of the family members of an individual suffering from SCD. Physicians should be alert and ask for genetic testing whenever SCD happens, beside a thorough autopsy. Genotype positive patients had a slightly higher risk for sudden cardiac death (p = 0.045, log-rank p = 0.031) confirming the results of previous investigators but not coming in total agreement with the recently published study by Maron^30^, where positive genotype was not associated with higher risk for SCD, supporting the emerging principle that HCM results from a diverse range of pathobiological mechanisms beyond a single molecular variant^34^. A better understanding of the factors that contribute to heterogeneous outcomes and lifetime disease burden in hypertrophic cardiomyopathy (HCM) is critically needed to improve patient management and outcomes.

It is noteworthy that the proportion of patients with a positive family history of HCM (24.4%) closely matched the proportion with a family history of sudden cardiac death (23.6%). Among the 53 patients with a family history of SCD, 33 (62.3%) also had a documented family history of HCM. This overlap suggests that familial inheritance was often recognized in families already known to be affected by the disease. However, in 37.7% of these cases, SCD was the only reported familial event, implying it may have been the first clinical manifestation of previously undiagnosed familial HCM. This raises the possibility of selection or reporting bias, whereby family history is more likely to be elicited or documented in the context of adverse outcomes such as SCD. Consequently, the true prevalence of familial HCM may be underestimated in patients without known SCD in their relatives. This limitation is particularly relevant in retrospective cohorts and highlights the importance of comprehensive family screening and genetic testing, which may help identify asymptomatic familial cases and improve risk stratification.

Recent studies^35^ have shown that up to 50% of genotype-positive individuals develop a phenotype of HCM over 15 years of follow-up independent of the age of screening. Family screening to identify affected relatives is therefore a key aspect of clinical care, allowing appropriate therapies to be instigated early and an opportunity to prevent disease-related complications. Surprisingly, in this cohort 17.2% (37/215) of the families investigated had a clinical phenotype of HCM at the initial clinical evaluation. From these 37 families, 34 had their index-proband genetically tested, where only in two of them there were no genetic variants detected. In another study^36^, HCM was identified in 37% of G + relatives at first clinical screening. However, in the study by Nielsen^37^ it is highlighted that family screening in HCM index patients who did not carry P/LP variants in recognized HCM genes identified few affected relatives with an apparent benign disease expression. The prevalence of relatives with HCM of genotype-negative index patients was only 5%. In a recently published study by Silajdzija^38^, of screened relatives 12% from gene-elusive families and 18% from genotype-positive families had phenotypic HCM at baseline. In our cohort only two gene-elusive families met the diagnosis of HCM at baseline. It may be considered premature to remove the recommendation for family screening in gene-elusive families, particularly given the finding that a diagnosis was still made in 12% of families (5% of relatives) in Nielsen’s study^37^, suggesting that family screening is still important for this patient group. In relatives of gene-elusive probands, Silajdzija et al.^38^ identified a baseline maximal wall thickness of ≥ 10 mm to be a strong predictive factor for development of HCM during 7 years follow-up, but further work is needed to identify which individuals will truly benefit.

## Limitations

Inherent limitations to retrospective, observational studies are survivor bias and the fact that inferences about causality cannot be made. Similarly, to the great majority of publications in HCM, this fact together with the low number of events limited the ability to test for an independent effect of the genotype and even compare the effects of the most common genes *MYBPC3* and *MYH7* in these HCM patients. Accordingly, the low number of events renders any multivariable analysis challenging and the results should be interpreted with caution.

Another limitation of this study is the inclusion of patients diagnosed with HCM many years prior to the study period, which may introduce survivor bias and affect the generalizability of the findings. Additionally, the absence of subgroup analyses based on diagnosis date limits the ability to fully assess the impact of this potential bias. However, the aim of the study was to describe the real-world clinical and genetic landscape of HCM patients currently under follow-up, inclusive of long-term survivors.

The predominance of sudden cardiac death (SCD)-related family history may also reflect a reporting or selection bias, whereby familial HCM is more likely to be identified and documented in the context of severe or fatal outcomes. This bias is particularly relevant in retrospective cohorts and may lead to underrecognition of familial cases in the absence of dramatic clinical presentations.

In this cohort, implantable cardioverter-defibrillators (ICDs) were implanted in 26.7% of patients, with appropriate ICD discharges observed in 10% of those implanted. The relatively high ICD implantation rate and frequency of appropriate therapies may similarly reflect selection bias, as patients referred to tertiary centers or included in retrospective registries tend to be at higher risk or already meet established criteria for device implantation. Furthermore, this pattern may indicate a shift in the study cohort toward more clinically advanced or higher-risk cases, potentially influenced by referral patterns, increased availability of genetic testing, or greater emphasis on early risk stratification in specialized centers.

An additional limitation of this study is that the “approved clinical genetic panel” used for testing varied over time and may not have included all currently known HCM-associated genes. Earlier versions of the panel had fewer genes compared to the more comprehensive, updated panels used in later years. The majority of tests (87 out of 172) were performed between 2018 and 2021 using an expanded panel. In contrast, earlier tests conducted between 2009 and 2017 used smaller panels. This variation in panel content over time may have influenced the detection rate of pathogenic variants. Consequently, some patients classified as genotype-negative in this study might be reclassified as genotype-positive if retested with the most current and extensive gene panels as it is highlighted in a previous publication^39^. This variability should be considered when interpreting the genetic findings and overall variant detection rates.

Variants of uncertain significance (VUS) remain a challenge in genetic interpretation due to their ambiguous clinical relevance. Some VUS variants may have since been reclassified as pathogenic; however, co-segregation analysis was not available in this study to support such reclassification. Sarcomeric VUS variants identified in patients with hypertrophic cardiomyopathy are typically disregarded in clinical practice due to this uncertainty. This cautious approach aligns with current guidelines, which recommend that VUS should not guide clinical management without further evidence. Nonetheless, excluding VUS variants may underestimate the true genetic contribution to HCM. Future studies incorporating segregation data and functional analyses are needed to clarify the role of these variants.

We believe this paper serves as a guide of the genotype status of the HCM patients in Sweden and there is an imperative need of multi-centre studies reporting phenotype and genotype of HCM patients for improvement of management and risk stratification.

## Conclusion

The majority of the recruited Swedish HCM patients had genetic testing. The percentage of positive results was almost 40% and the distribution amongst causal genes are similar to published populations. Importantly, the genotype-positive patient cohort has distinct imaging characteristics and family history, seems to be at increased risk for sudden cardiac death and has more affected relatives with HCM at first clinical screening. However, G + status should not be used to dictate clinical management or predict outcome in HCM as this present Swedish HCM cohort demonstrated no difference in mortality, heart failure, and malignant arrhythmias related to either genotype‐positive or genotype‐negative status even if there was low number of events in this HCM cohort. Therefore, the data underscore an important principle in HCM in which G − status does not confer immunity from adverse complications.

## Supplementary Information

Supplementary Information.

## Data Availability

The datasets generated and/or analysed during the current study are not publicly available due to patient privacy protections under the European General Data Protection Regulation (GDPR), but are available from the corresponding author on reasonable request.
