# Supplementary material for: Genetic determinants of the phenotype in a Swedish cohort of patients with hypertrophic cardiomyopathy
Source: Sci Rep. 2025 Nov 10;15:39357. doi: 10.1038/s41598-025-27238-9 (PMC12602694; doi:10.1038/s41598-025-27238-9)
Supplement: Supplementary file 1 — Supplementary Information. [file 41598_2025_27238_MOESM1_ESM.pdf]

**Supplementary Tables**

| | **N (%)** |
|---|---|
| Hypertension | 106 (63.5) |
| Storage Disease | 5 (3) |
| Syndromes (Microdeletion, Noonan, Leopard) | 4 (2.4) |
| Steroids/Drugs | 2 (1.2) |
| Amyloidosis (TTR+AL) | 13 + 3 (9.6) |
| Non-compaction/Dilated Cardiomyopathies | 19 (11.4) |
| Neuromuscular Diseases (Ataxia) | 4 (2.4) |
| Athlete´s Heart | 3 (1.8) |
| Subvalvular Aortic Membrane | 3 (1.8) |
| Aortic Stenosis | 4 (2.4) |
| Mitocondrial disease (MELAS) | 1 (0.6) |
| Totally | 167 |

**Supplementary Table 1.** Underlying diagnoses of different aetiologies of HCM
TTR: Transthyretin amyloidosis, AL: amyloid light chain amyloidosis, MELAS: Mitochondrial encephalomyopathy with lactic acidosis and stroke-like episodes syndrome

| GENETIC VARIANTS |
|---|
| ***MYBPC3*** |
| NM_000256.3(*MYBPC3*):c.710A>C |
| NM_000256.3(*MYBPC3*):c.2490dup |
| NM_000256.3(*MYBPC3*):c.833del |
| NM_000256.3(*MYBPC3*):c.3697C>T |
| Homozygous NM_000256.3(*MYBPC3*):c.2618C>T |
| NM_000256.3(*MYBPC3)*:c.2864_2865del |
| NM_000256.3(*MYBPC3*):c.3330+5G>C |
| NM_000256.3(*MYBPC3*):c.2373dup |
| NM_000256.3(*MYBPC3)*:c.3190+2T>G |
| NM_000256.3(*MYBPC3*):c.3190+5G>A |
| NM_000256.3(*MYBPC3*):c.2556_2557delinsTCT |
| NM_000256.3(*MYBPC3)*:c.553_562del |
| NM_000256.3(*MYBPC3*):c.506-1G>A |
| NM_000256.3(*MYBPC3*):c.3043dup |
| NM_000256.3(*MYBPC3*):c.1957_1969delinsATGCT |
| NM_000256.3(*MYBPC3*):c.3257G>A |
| NM_000256.3(*MYBPC3*):c.3330+2T>C |
| ***MYH7*** |
| NM_000257.4(*MYH7*):c.4130C>T |
| NM_000257.4(*MYH7*):c.428G>A |
| NM_000257.4(*MYH7*):c.1988G>A |
| NM_000257.4(*MYH7*):c.427C>T |
| NM_000257.4(*MYH7*):c.2609G>A |
| NM_000257.4(*MYH7)*:c.1063G>A |
| NM_000257.4(*MYH7*):c.2100G>T |
| NM_000257.4(*MYH7*):c.2770G>A |
| NM_000257.4(*MYH7*):c.3158G>A |
| NM_000257.4(*MYH7*):c.5135G>A |
| NM_000257.4(*MYH7*):c.1207C>G |
| NM_000257.4(*MYH7*):c.5136G>A |
| NM_000257.4(*MYH7*):c.5287G>A |
| ***ACTC1*** |
| NM_005159.5(*ACTC1*):c.301G>A |
| ***MYL2*** |
| NM_000432.4(*MYL2*):c.173G>A |
| NM_000432.4(*MYL2*):c.64G>A |
| ***ALPK3*** |
| NM_020778.5(*ALPK3*):c.903del |
| ***FLNC*** |
| NM_001458.5(*FLNC*):c.539A>G |

**Supplementary Table2.**

The genetic variants observed in the HCM patients recruited from the Southeast region in Sweden from 2011-2021. The analytical report was issued by an accredited laboratory according to internationally recognised standards, and the variants were determined

pathogenic or likely pathogenic at the time for the analyses. Variants were assessed according to American College of Medical Genetics and Genomics (ACMG) guidelines. Some of the same P/LP variants in *MYBPC3* and *MYH7* genes were detected in several patients. All of these variants were detected in heterozygous state, beside the variant NM_000256.3(*MYBPC3*):c.2618C>T that was found in homozygous state

| Patient | NM_Gene_Variant VUS | Category |
|---|---|---|
| 1 | NM_000256.3 *MYBPC3* c.2441_2443del | Sarcomeric |
| 2 | NM_005691.3 *ABCC9* c.200C>T | Other |
| 3 | NM_133378.4 *TTN* c.42850C>T | Cytoskeletal |
| 4 | NM_000364.3 *TNNT2* c.853C>T | Sarcomeric |
| 5 | NM_003476.3 *CSRP3* c.10T>C | Sarcomeric |
| 6 | NM_018480.3 *TMEM126B* c.545G>T | Other |
| 7 | NM_004415.3 *DSP* c.5593A>T | Cytoskeletal |
| 7 | NM_004369.3 *COL6A3* c.6422C>A | Other |
| 7 | NM_004281.3 *BAG3* c.72C>A | Cytoskeletal |
| 7 | NM_004006.2 *DMD* c.2635C>T | Cytoskeletal |
| 8 | NM_000364.3 *TNNT2* c.762G>T | Sarcomeric |
| 9 | NM_001035.2 *RYR2* c.48+8C>G | Other |
| 9 | NM_000218.2 *KCNQ1* c.1378G>A | Other |
| 10 | NM_002471.4 *MYH6* c.161G>A | Sarcomeric |
| 11 | NM_000257.3 *MYH7* c.1625A>G | Sarcomeric |
| 12 | NM_000256.3 *MYBPC3* c.3392T>C | Sarcomeric |
| 12 | NM_033118.3 *MYLK2* c.4G>A | Sarcomeric |
| 12 | NM_001458.3 *FLNC* c.4877T>C | Cytoskeletal |
| 13 | NM_000337.3 *SGCD* c.593G>A | Cytoskeletal |
| 14 | NM_003476.3 *CSRP3* c.10T>C | Sarcomeric |
| 15 | NM_000256.3 *MYBPC3* c.2497G>A | Sarcomeric |
| 16 | NM_000256.3 *MYBPC3* c.3763G>A | Sarcomeric |
| 17 | NM_138395.3 *MYPN* c.3833G>A | Cytoskeletal |
| 18 | NM_001256267.1 *MYPN* c.2228C>T | Cytoskeletal |
| 19 | NM_001008844.2*DSP* c.1996A>G | Cytoskeletal |
| 20 | NM_003476.3 *CSRP3* c.109T>C | Sarcomeric |
| 20 | NM_004572.3 *PKP2* c.184C>A | Cytoskeletal |
| 21 | NM_000257.3 *MYH7* c.2397G>A | Sarcomeric |
| 21 | NM_000337.5 *SGCD* c.593G>A | Cytoskeletal |
| 22 | NM_144573.3 *NEXN* c.917G>A | Cytoskeletal |
| 22 | NM_000724.3 *CACNB2* c.590C>T | Other |
| 22 | NM_001613.3 *ACTA2* c.977C>A | Sarcomeric |
| 22 | NM_001267550.2 *TTN* c.43010G>A | Cytoskeletal |
| 23 | NM_001103.4 *ACTN2* c.8893G>A | Sarcomeric |
| 23 | NM_022114.4 *NEBL* c.2761G>C | Cytoskeletal |
| 24 | NM_004100.4 *EYA4* c.899C>A | Other |
| 24 | NM_002471.3 *MYH6* c.3784C>T | Sarcomeric |
| 25 | NM_000256.3 *MYBPC3* c.405A>G | Sarcomeric |
| 26 | NM_138395.3 *MYPN* c.1046C>A | Cytoskeletal |

| Patient | NM_Gene_Variant VUS | Category |
|---|---|---|
| 27 | NM_000257.3 *MYH7* c.4657C>G | Sarcomeric |
| 28 | NM_001267550.2 *TTN* c.28031-1G>A | Cytoskeletal |
| 29 | NM_000256.3 *MYBPC3* c.716G>A | Sarcomeric |
| 29 | NM_000363.4 *TNNI3* c.428C>A | Sarcomeric |
| 30 | NM_004281.3 *BAG3* c.554C>T | Cytoskeletal |
| 31 | NM_002471.3 *MYH6* c.5485G>C | Sarcomeric |
| 31 | NM_001035.2 *RYR2* c.8688G>A | Other |
| 32 | NM_001281740.2 *FHOD3* c.1004C>A | Cytoskeletal |
| 33 | NM_001458.3 *FLNC* c.5727_5729del | Cytoskeletal |

**Supplementary Table3.**

This table presents a comprehensive summary of the variants of uncertain significance (VUS) identified in 33 patients with hypertrophic cardiomyopathy (HCM). Several patients carry multiple VUS across different genes. Each variant is listed alongside its NM reference sequence, gene name (italicized), and specific nucleotide change. Genes are categorized into sarcomeric, cytoskeletal, and other groups based on their primary biological roles. All variants remain classified as VUS according to ACMG guidelines, indicating that their pathogenicity and contribution to the HCM phenotype are currently unclear. As a result, they were excluded from the main analysis. This highlights the need for further functional studies and clinical data to clarify their relevance and potential implications on patient management.

- **Sarcomeric genes**: *MYBPC3, MYH7, MYH6, TNNT2, TNNI3, CSRP3, ACTN2, MYL2, MYLK2*
- **Cytoskeletal genes**: *MYPN, FLNC, DSP, BAG3, TTN, SGCD, PKP2, FHOD3, NEBL*
- **Other / ion channel genes**: *TMEM126B (*mitochondrial gene*), EYA4* (regulatory gene)*, ABCC9, KCNQ1, RYR2, CACNB2*
